# Casein kinase 2 attenuates brain injury induced by intracerebral hemorrhage via regulation of NR2B phosphorylation

**DOI:** 10.3389/fncel.2022.911973

**Published:** 2022-07-19

**Authors:** Zhimin Sun, Qiyao Li, Xiaopeng Li, Yunpeng Shi, Chengrui Nan, Qianxu Jin, Xiaoyan Wang, Yayu Zhuo, Zongmao Zhao

**Affiliations:** ^1^Department of Neurosurgery, The Second Hospital of Hebei Medical University, Shijiazhuang, China; ^2^Department of Neurosurgery, The Third Hospital of Shijiazhuang City, Shijiazhuang, China; ^3^Department of Neurosurgery, The First Hospital of Handan City, Handan, China; ^4^Department of Neurosurgery, Hebei General Hospital, Shijiazhuang, China

**Keywords:** intracerebral hemorrhage, NR2B, apoptosis, inflammation, oxidative stress

## Abstract

**Objective:**

Intracerebral hemorrhage (ICH) is a common cerebrovascular disease with high incidence, disability, and mortality. Casein kinase 2 (CK2) is a serine/threonine kinase with hundreds of identified substrates and plays an important role in many diseases. This study aimed to explore whether CK2 plays protective roles in ICH-induced neuronal apoptosis, inflammation, and oxidative stress through regulation NR2B phosphorylation.

**Methods:**

CK2 expression level of brain tissues taken from ICH patients was determined by immunoblotting. Neurons from embryonic rat and astrocytes from newborn rats were cultured and treated by Hemoglobin chloride (Hemin). The proliferation of astrocytes, the apoptosis and oxidative stress of neurons and the inflammatory factors of astrocytes were detected. CK2 expression was determined in ICH model rats. The effects of CK2 overexpression plasmid (pc-CK2) on neurobehavioral defects and brain water content in ICH rats were observed.

**Results:**

CK2 expression in ICH patients was down-regulated. Overexpression of CK2 promoted the astrocyte proliferation, inhibited neuronal apoptosis, and reduced astrocyte-mediated inflammation. *N*-methyl-D-aspartate receptor 2B (NR2B) reversed the effects of pc-CK2 on neurons and astrocytes. CK2 phosphorylated NR2B at the S1480 site, down-regulated the expression of NR2B and interfered with the interaction between NR2B and postsynaptic density protein 95 (PSD95). *In vivo* experiments showed that the expression of CK2 decreased and the expression of NR2B increased in ICH rats. Furthermore, pc-CK2 attenuated neurobehavioral defects, brain water content and neuronal damage in ICH rats.

**Conclusion:**

CK2 phosphorylated NR2B, down-regulated the expression of NR2B, interfered with the interaction between NR2B and PSD95, alleviated inflammatory reactions, inhibited neuronal apoptosis and oxidative stress after ICH. CK2 and NR2B may be new potential therapeutic targets for the treatment of ICH. However, the limitation of this study is that we only investigated the regulation of NR2B by CK2.

## Introduction

Intracerebral hemorrhage (ICH) is caused by non-traumatic brain parenchymal vascular rupture and accounts for about 15 ∼ 20% of the total stroke cases. The mortality of ICH at the early stage is very high, and most survivors have severe sequelae such as motor cognitive and language impairment (Chen et al., 2018; Wilkinson et al., 2018; Marques et al., 2019). The etiology of ICH mainly includes cerebrovascular dysfunctions during pathological conditions such as hypertension, hyperlipidemia, diabetes, aging, smoking and drinking, etc. ICH induces a huge economic and social burden due to the increased incidence in young patients (Qureshi et al., 2001; Whiteley et al., 2012). However, there is no effective treatment for ICH with high mortality and disability (Murthy et al., 2017; Imai et al., 2021).

Clinical and animal studies have shown that ICH is closely related to inflammation, oxidative stress, neuronal apoptosis and blood brain barrier (BBB) damage after hematoma formation (Lee et al., 2017; Lan et al., 2019; Selim and Norton, 2020). Rapid accumulation of blood in the brain parenchyma, elevated intracranial pressure, and damaged neurons and glial cells may lead to an increase in neurotransmitter release, neuron membrane depolarization, and mitochondrial dysfunction (MacLellan et al., 2008). Subsequently, along with coagulation reaction, thrombin generation and decomposition of hemoglobin, glial cells are activated, which releases pro-inflammatory factors and causes excessive inflammatory response (Selim and Norton, 2020). At the same time, an increase in BBB permeability and blood-derived cerebral edema may also occur. All of these factors lead to serious neurological defects in patients, such as sensorimotor disorders, consciousness disorders, and even respiratory dysfunction (Zhu et al., 2018). These events lead to the deterioration of the prognosis of the ICH. In addition, the interruption of cerebral blood flow during cerebral hemorrhage result in severe neuronal damage. In the early stage of hemorrhage, the apoptosis proteins of caspase family in nerve cells of the central ischemic area are activated significantly to trigger apoptosis and irreversible damage of neurons (Sekerdag et al., 2018). Up to date, the main approach for clinical prevention and treatment of ICH is controlling hypertension and other risk factors. Thus, it is urgent to find new targets that play a vital role in the pathological mechanism of ICH.

Casein kinase2 (CK2) is a highly conserved phosphorylated serine/threonine kinase widely distributed in various eukaryotes with a variety of physiological functions (Filhol et al., 2004). CK2 is a hetero-tetramer composed of 2 α catalytic subunits and 2 β regulatory subunits. CK2 plays an important role in the regulation of hundreds of identified substrates and can use both ATP and GTP as phosphate donor kinases (Bolanos-Garcia et al., 2006; Dominguez et al., 2011). Studies have shown that mice with global knockout CK2α or CK2β cannot survive in the second trimester of pregnancy (Moreno-Romero et al., 2008; Landesman-Bollag et al., 2011; Abi Nahed et al., 2020), suggesting that CK2 plays a key role in their development. In addition, CK2 is involved in important biological processes such as circadian rhythm (Allada and Meissner, 2005), inflammation (Singh and Ramji, 2008), and cancer (Seldin et al., 2008; Dominguez et al., 2009). CK2 also plays an important role in cell processes, including cell proliferation and growth (Litchfield, 2003), cell survival (Ahmad et al., 2008), cell cycle progression (Padilla-Benavides et al., 2020), cell apoptosis and transcription (Meggio and Pinna, 2003; St-Denis and Litchfield, 2009), cell differentiation (Guerra and Issinger, 1999; Dietz et al., 2009), cell metabolism (Kramerov et al., 2008), etc. CK2 increases *N*-methyl-D-aspartate receptor (NMDAR) activity by upregulating *N*-methyl-D-aspartate receptor 2A (NR2A) and phosphorylating NR2B on S1480 at presynaptic and postsynaptic sites in the hypothalamus of spontaneous hypertension rat (SHR), which is a key factor in paraventricular nucleus of hypothalamus (PVN) presympathetic neuron over-excitability in hypertension (Ye et al., 2012). In ischemic brain injury, reduced CK2 expression induces a transfer of P47phox, P67phox and Rac1 to the cell membrane, resulting in activation of nicotinamide adenine dinucleotide phosphate (NADPH) oxidase, which in turn increases the production of reactive oxygen species (ROSs) and the up-regulation of NOX, mediating the death of neurons (Kim et al., 2009). However, the changes of CK2 mRNA and protein levels and the role of CK2 after ICH remain unclear.

Brain hemorrhage leads to a rapid increase in glutamate levels, which activates NMDAR, thereby damaging neurons. NMDAR plays a crucial role in excitatory synaptic transmission and plasticity. The functional NMDAR usually consists of two NR1 subunits and two NR2 (NR2A-D) subunits (Lee et al., 2014). The NR2B subunit determines many of the functional properties of NMDAR. Multiple amino acid sites at the end of NR2B that can be phosphorylated play different roles in NMDAR-mediated injury. Phosphorylation of NR2B at the S1303 site exacerbates ischemia-induced neuronal death (Tu et al., 2010). Phosphorylation at the Tyr1472 site interferes with the binding of NR2B and adapter2 (AP2) protein leading to increased expression of NR2B on the cell membrane (Lavezzari et al., 2003). The phosphorylation of NR2B at S1480 site affects the binding of NR2B to PZD domain, which promotes the intracellular transfer of NMDA receptor and down-regulates the expression of NR2B (Chung et al., 2004). It has been shown that the NR2B subunit plays a key role in promoting neuronal death under ischemic conditions (Vieira et al., 2016). In addition, the PDZ ligand on the NR2B subunit binds to the PDZ structural domain of the scaffolding protein (PSD-95) to form the NR2B-PSD95 complex, which regulates downstream synaptic signaling and participates in NMDA receptor-mediated neuroexcitation and neurotoxicity (Lai et al., 2011). Therefore, in this study, we determined whether NR2B, NR2B phosphorylation and the NR2B-PSD95 complex play an important role in the regulation of cerebral hemorrhage by CK2.

This study aimed to explore whether CK2 plays protective roles in ICH-induced neuronal apoptosis, inflammation, and oxidative stress through regulation NR2B phosphorylation. First, we investigated whether CK2 is involved in the progression of ICH injury using patient samples, rat ICH model and cultured primary neurons and astrocytes. The potential neuroprotective mechanisms of CK2 *in vitro* and *in vivo* were further explored. In this study, we found that CK2 reduced inflammatory response, neuronal apoptosis, and oxidative stress levels after ICH through regulating NR2B phosphorylation, and subsequently changing NR2B expression and the level of NR2B-PSD95 complex. To our knowledge, this is the first study showing that CK2 protects brain tissue from damage caused by ICH.

## Materials and methods

### Patient tissue sample

In this study, the brain tissue specimens and clinical data of 10 healthy controls and 10 patients with ICH were collected and analyzed. All patients in this study were informed and signed consents according to the guidelines approved by the Ethics Committee of the second Hospital of Hebei Medical University. The inclusion criteria of the ICH group were 18–80 years old, without gender restriction, in accordance with the diagnostic criteria of ICH confirmed by CT, and no other autoimmune diseases. The National Institutes of Health Stroke scale (NIHSS) was 11.05 ± 3.46. The control group had no cerebral hemorrhagic disease and no history of neurological or neuropsychiatric diseases. The specimens were collected with the informed consent of the patient’s family. After the brain tissue was removed, it was frozen in liquid nitrogen immediately. The brain tissues of all patients in the control group and cerebral hemorrhage group were used for mRNA and protein levels analysis.

### Cultured neurons and astrocytes *in vitro*

On the 20th day of pregnancy, the fetal rats were taken by laparotomy, their brain tissue was taken out and put in ice-cold Hank’s balanced salt solutions. Their cortex was collected and cut into small pieces of 1 × 1 × 3 mm. Then, the solution containing trimmed brain tissue blocks was shook at 37°C for 15 min, and the medium containing 10% fetal bovine serum (FBS) was added to terminate digestion. The tissue suspension was centrifuged at 1000 rpm for 2 min and the supernatant was removed and the pellets were re-suspended in a complete culture medium consisting of DMEM/high glucose, 10% FBS (Gibco, United States) and 1% penicillin-streptomycin solution (HyClone, United States). The neurons were placed in a 96-well plate coated with poly-lysine with a cell density of 5 × 10^5^/ml. The cells were incubated at 37°C and 5% CO_2_ for 4 h and then the complete medium was replaced with neural basic medium (Thermo, 2110304, United States), and half of the medium was changed with fresh medium every 3 days. In order to culture astrocytes, newborn rats within 24 h were anesthetized with isoflurane and the brain was removed under aseptic condition and placed in CMF-HBSS. Under anatomical microscope, the cerebral hemisphere was removed, meninges and blood vessels and other connective tissue were removed, and the tissue was cut into small pieces as finely as possible. These small pieces were immersed in trypsin solution to which DNase had been added. The solution was cultured at 37°C for 5 min, and the solution was ground repeatedly with a pipette until most of the chunks disappeared. The cell suspension was collected in the colloid medium (sciencell, 1801, United States) through the cell filter. The cells were centrifuged with 1,000 *g* for 2 min, the supernatant was discarded, and the cell particles were re-suspended in colloid medium. 2.5 × 10^6^ cells were cultured in T25cm^2^ flask and changed to fresh medium 24 h later. The culture medium was changed every 3 days. After 7–10 days, the astrocytes covered the bottom of the bottle. The bottle was shaken, the supernatant was removed, and the cells were passaged growing at the bottom of the culture bottle. The neurons and astrocytes cultured in 96-well plates were treated with different concentrations of hemoglobin (Hemin) (Meilunbio, MB4829, Dalian, China). After 24 h, the cell viability was determined by CCK-8. The 20 μmol/L of Hemin was selected to establish the cell model of ICH *in vitro* (Levy et al., 2002). Follow-up experiments were performed at 24 h after the establishment of cell ICH model by Hemin.

### CCK8 assay

According to the instructions of CCK kit (ZOMANBIO, ZP328, Beijing, China), CCK-8 assay was carried out to determine the cell proliferation ability or inhibition rate. The cells were cultured in 96-well plate and treated respectively by each group. The blank groups were used as controls. At the end of the treatment, the culture medium was replaced with 10% CCK-8 solution and incubated for 2 h, then the absorbance at 450 nm was measured and the cell viability of each group was calculated.

### Western blot

The total protein was extracted from the striatum tissue around the hematoma and the cells of each group, and the concentration was determined by BCA. SDS-PAGE gel was made by dispensing gel kit (ZOMANBIO, ZD304A-1, Beijing, China). 20μg protein of each sample was loaded on SDS-PAGE. The protein was separated by electrophoresis and transferred to PVDF membrane (Roche, 03010040001, Switzerland). 5% milk blocked non-specific antigen for 1.5 h, and the membrane was incubated with appropriate diluted primary antibody overnight at 4°C. The main antibodies were used as follows: CK2 (Abcam, ab76040, 1:2000), NR2B (Cell Signaling, #14544, 1:500), NR2B (Tyr 1472) (Cell Signaling, #4208, 1:500), NR2B (phospho S1303) (Abcam, ab81271, 1:500), NR2B (phospho S1480) (Abcam, ab73014, 1:500), Caspase3 (GeneTex, GTX86952, 1:500), GAPDH (ABclonal, AC001, 1:50000). The PVDF membrane was incubated with fluorescent secondary antibody (ROCKLAND,611-145-002) at room temperature for 2 h the next day, and the Odyssey infrared laser scanning imaging system (LI-COR, United States) was used to image and store the PVDF membrane. The Photoshop software was used to measure the grayscale value of the stripe. The average optical density of the target protein was calculated with the gray value of GAPDH as the internal reference.

### Relative CK2 activity measurement

The CycLex CK2 Kinase Assay/Inhibitor Screening Kit (MBL International Cat#CY-1170, Japan) was used to measure CK2 activity in brain lysates and cell lysates according to the manufacturer’s instructions.

### Cell transfection

To transfect CK2 and NR2B, primary cortical neurons and astrocytes were cultured in a 24-well petri dish. Gene overexpression lentivirus was purchased from Hanheng Biological Company (Shanghai, China). According to the manual of lentivirus, MOI = 200 was taken, and the corresponding volume of virus solution (volume of virus solution = MOI × number of cells/virus titer) was added, and the volume of culture medium was made up after 4 h. After 24 h, the culture medium was changed to complete medium and continued culture. Puromycin screening was carried out 48 h later, and 48 h after drug addition, puromycin was removed and fresh medium was replaced. When these petri dishes were full of cells, follow-up experiments were carried out.

### RT-PCR

Total RNA was extracted using RNA extraction kit (Takara, Dalian, China) and the concentration of RNA was measured. Reverse transcription of RNA into cDNA using PrimeScript RT kit (Takara). cDNA was used as a template and the instructions provided by SYBR^®^ Premix Ex TapTM II Kit (Takara) were followed for RT-qPCR. With GAPDH as the internal reference, the relative expression of the gene was calculated by ^–ΔΔCt^ method.

### Transferase-mediated nick end labeling assay

According to the instructions of Meilun One Step transferase-mediated nick end labeling (TUNEL) Apoptosis Assay Kit (TRITC) (Meilunbio MA0224, Dalian, China), after the neurons of each group were fixed and permeated, the neurons were exposed to TUNEL detection solution for 1 h under the condition of avoiding light at 37°C. After PBS washing, the neurons were stained with DAPI (Sigma, D9542, United States) for 15 min at room temperature, and the photos were taken under fluorescence microscope (Leica, Germany). TUNEL staining showed that the nucleus was red and DAPI staining showed that the nucleus was blue. The percentage of TUNEL/DAPI was measured by ImageJ software to quantify apoptosis.

### Enzyme-linked immunosorbent assay (ELISA)

The culture supernatant of astrocytes in each group was collected and the precipitate was removed by centrifugation. According to the ELISA detection kit (ABclonal, RK00029; RK00020, China), the levels of tumor necrosis factor (TNF)-α and interleukin (IL)-6 in the supernatant were detected.

### Oxidative stress detection

In order to detect reactive oxygen species (ROS), neuronal cells were cultured in 96-well petri dishes at 37? for 30 min, and Highly Sensitive DCFH-DA Dye (ROS Assay Kit -Highly Sensitive DCFH-DA-Dye, DOjinDO, R252) was added to each petri dish. After HBSS cleaned the petri dish, the fluorescence microscope (Leica, Germany) was used to take pictures. The fluorescence intensity and the relative content of ROS were measured by ImageJ software. In order to detect glutathione (GSH), superoxide dismutase (SOD) and oxidation marker malondialdehyde (MDA), the culture supernatants of neurons in each group were collected. The contents of GSH, MDA and SOD were detected by kit (Jiangsu Nanjing Jiancheng Institute of Biological Engineering, A006-2-1; A003-4-1; A001-3). Each reaction solution was added to a 96-well plate, and the absorbance at 405 nm was measured on a microplate reader, and the GSH content was calculated. The absorbance value at 530 nm was measured to calculate the MDA content. The absorbance at 450 nm was measured and the content of SOD was calculated. The experiment was repeated three times.

### Co-immunoprecipitation

In order to determine the effects of Hemin and pc-CK2 on the interaction between NR2B and PSD95, we collected brain samples and samples of neurons and astrocytes from each group of rats for experiments. After homogenizing the samples, cool low osmotic buffers (1 mmol/L CaCl2, 20 mmol/L Tris, pH 7.4, 1 mmol/L MgCl2 and protease inhibitor mixture) were prepared for membrane preparation. The supernatant was centrifuged at 21,000 *g* for 30 min. The particles were re-suspended and dissolved in immunoprecipitation buffer (mixture of protease inhibitors, 0.5%NP-40, 250 mmol/L NaCl, 50 mmol/L Tris, pH 7.4), and the soluble fraction was pre-combined with protein G beads overnight at 4°C to pre-bind to rat anti-NR2B antibodies(Cell Signaling, #14544, 1:50). As a control, protein G beads were bound to rat immunoglobulin G (IgG) in advance. The immunoprecipitation samples were washed for three times, and then immunoblotting was performed with anti-PSD95 antibody (HUABIO, ET1602-20, 1:3000).

### Animal and intracerebral hemorrhage model

The Sprague-Dawley (SD) male rats (age 8–10 weeks, weight 280–320 g) were obtained from the *Invivo* Biotech Co. td (Shijiazhuang, China). The rats were randomly placed in cages and fed with normal food, and the temperature (23 ± 1°C) and humidity (55 ± 2%) were controlled. All animal uses and research programs have been approved by the Ethics Committee of the Second Affiliated Hospital of Hebei Medical University. These rats were divided into Sham group, Sham + PC-CK2 group, ICH group, ICH + PC-NC group and ICH + PC-CK2 group. According to the study of GaryA. Rosenberg, MD (Rosenberg et al., 1990), the rat model of ICH was established by injecting collagenase into the striatum. Rats were anesthetized by intraperitoneal injection of 1% pentobarbital sodium (40 mg/kg) and fixed on the stereotactic frame (NeuroStar company, Germany). A skull hole of 1 mm in diameter was drilled at the posterior 0.24 mm of the anterior fontanelle and 3 mm on the right side of the midline, and 1 μ l collagenase (0.3 U/μl, type IV, Sigma-Aldrich, V900893) was injected into the right striatum (0.2 μ l/min) through the PE tube (62204, Shenzhen RWD Life Technology Co., Ltd.) with a microinjection pump (KDS310 American KDS Co., Ltd.). During the operation, the rats were placed on a heating blanket to maintain their body temperature at 37 ± 0.5°C. The animals in the sham operation group were only injected with 1 μl normal saline without collagenase IV. Three days before modeling, the right striatum of rats in Sham + pc-CK2 group, ICH + pc-CK2 group and ICH + pc-NC group were microinjected with lentivirus overexpression vectors pcDNA-CK2 and pcDNA NC (Hanheng Biology, Shanghai, China). The lentivirus titer was 1 × 108 U/mL, the injection dose was 0.1 ml, and the needle was retained for 5 min after injection.

### Neurobehavioral score

Forelimb placement test, corner turn test and Bederson score were used to evaluate the neurological function within 10 days after the establishment of ICH model by collagenase. In the forelimb placement test, when the beard of the rats was rubbed with the edge of the table corner, the healthy rats could immediately put the ipsilateral forelimb above the table corner, while the contralateral motor function of ICH rats was impaired and might not be able to complete this action. Each animal was tested 10 times, and the percentage of times of correct placement of the forelimb was recorded (Hartman et al., 2009). During Corner Turn Test, the rats were placed in a 30-degree corner, and in order to get away from the corner, the rats could turn left or right, which was recorded, including only fully upright turns along either wall (that is, excluding abdominal pleats or horizontal turns). Repeated this 10 times to calculate the percentage of the right turn (Hua et al., 2002). Another behavioral test involved was the Bederson score, which was divided into four grades: no neurological impairment, (1) the contralateral forepaw could not be fully extended in the tail suspension test, (2) the forelimb’s ability to resist contralateral thrust decreased, and (3) turned the contralateral circle (Bederson et al., 1986).

### Determination of brain water content

Rats were anesthetized with pentobarbital (60 mg/kg) and the brains were dissected quickly. Three samples were obtained from the brain: ipsilateral hemisphere, contralateral hemisphere and cerebellum. The brain samples were immediately weighed on the electronic analytical balance (Sartorius, China) to obtain wet weight. The brain sample was then dried at 100°C for 24 h to obtain dry weight. The calculation formula is as follows: (wet weight-dry weight)/wet weight × 100%.

### Statistical analysis

All the results came from at least three independent experiments. Statistical analysis was carried out with GraphPad Prism8 software. To determine the single comparison between the two groups, Student’s *t*-test, One-way analysis of variance or Two-way ANOVA was used to compare the differences between multiple groups. All data are expressed by mean ± standard error, and a *p*-value of less than 0.05 was considered to be statistically significant.

## Results

### CK2 expression level was decreased in patients with intracerebral hemorrhage

**FIGURE 1:**
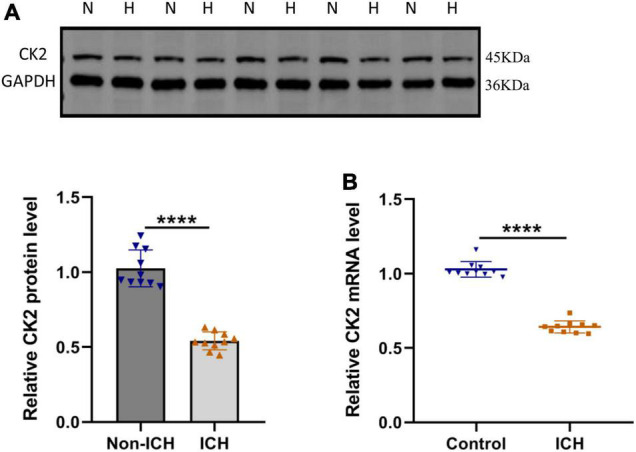
CK2 was downregulated in patients with intracerebral hemorrhage. **(A)** Representative blots and summary data show CK2 protein level in the brain tissues from ICH patients or non-hemorrhagic controls (*n* = 10). **(B)** Relative mRNA levels of CK2 were analyzed (*n* = 10). The experiment was repeated three times independently. Data were shown as mean ± SD and he values between groups were compared by Student’s *t*-test. ****P < 0.0001.

In order to determine the CK2 expression levels in ICH patients, we measured the CK2 protein and mRNA levels in the brain tissues of 10 ICH patients. CK2 protein and mRNA levels were significantly lower in ICH tissues than those in control tissues (*P* < 0.0001; Figures 1A,B).

### Changes of CK2 activity and protein level and NR2B phosphorylation level in brain tissue of intracerebral hemorrhage rats

**FIGURE 2:**
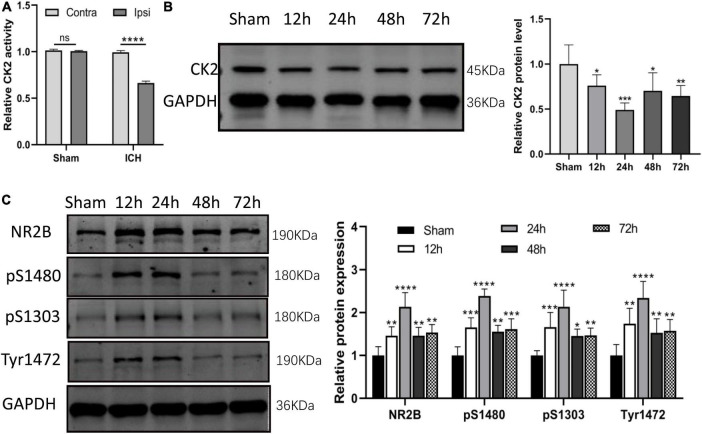
CK2 was downregulated in the brain tissues of ICH rats. **(A)** The CK2 activity in ipsilateral and contralateral hemisphere of sham and ICH groups (*n* = 3). **(B)** Expression of CK2 were determined 12, 24, 48, and 72 h after ICH in rats using Western blot. **(C)** Expression of NR2B, NR2B phosphorylation sites S1480, S1303, and Tyr1472 were determined 12, 24, 48, and 72 h after ICH in rats using Western blot (*n* = 6). Data were expressed as mean ± SD. *p < 0.05, **p < 0.01, ***p < 0.001, ****p < 0.0001.

In ICH rats induced by injection of collagenase IV into the striatum, CK2 activity was measured in tissue taken from damaged cerebral cortex and striatum and undamaged contralateral hemisphere at 24 h after the establishment of ICH model by collagenase. The CK2 activity of the two hemispheres in the sham operation control group was similar, but compared with the contralateral hemisphere, the CK2 activity in the ipsilateral hemisphere decreased significantly after ICH (*P* < 0.0001; Figure 2A). Furthermore, the levels of CK2 protein in the tissue around the hematoma was measured at 12, 24, 48, and 72 h after the establishment of ICH model by collagenase. The results showed that compared with the control group, CK2 protein was decreased after hemorrhage, especially at 24 h (*P* < 0.001; Figure 2B). The level of NR2B and its phosphorylation sites increased after ICH, especially at 24 h (*P* < 0.0001; Figure 2C).

### Overexpression of CK2 alleviates brain injury in intracerebral hemorrhage rats

**FIGURE 3:**
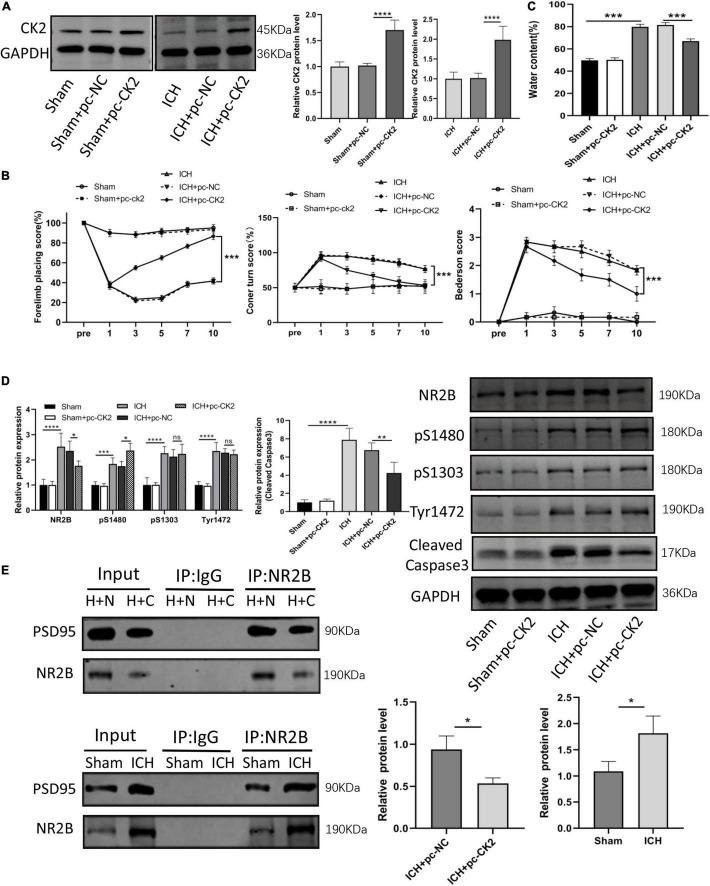
Overexpression of CK2 alleviated brain injury in ICH rats. pcDNA-CK2 and pcDNA-NC were transfected into ICH rats. **(A)** Expression of CK2 was detected using Western blot (*n* = 6). **(B)** Neurologic score was determined by the Forelimb placing test, corner turn test and Bederson score. **(C)** Analysis of brain edema. **(D)** Expression of cleaved caspase3, NR2B, NR2B phosphorylation sites S1480, S1303, and Tyr1472 were determined in rats using Western blot (*n* = 6). **(E)** Representative gel images and quantification of co-immunoprecipitation (co-IP) show the level of NR2B-PSD95 complex in rat brain tissues (*n* = 3). Data were expressed as mean ± standard deviation. *p < 0.05, **p < 0.01, ***p < 0.001, ****p < 0.0001.

We further verified the effect of overexpression of CK2 on neurological function ICH rats. Figure 3A verified that CK2 was overexpressed by pcCK2 viral vector (*P* < 0.0001). The neurological function score was determined by Bederson scale, forelimb placement, and rotation angle tests. ICH + pc-NC rats showed a severe neurological impairment, which indicated that cortical and striatal functions were impaired and motor and sensory coordination was weakened. Pc-CK2 treatment significantly alleviated neurobehavioral impairment in ICH rats (all *P* < 0.001; Figure 3B). Compared with sham-operated rats, the brain water content in ICH group was increased (*P* < 0.001; Figure 3C), while pc-CK2 treatment decreased brain water content (*P* < 0.001; Figure 3C). Western Blot was used to detect the level of NR2B and its phosphorylation sites proteins, compared with ICH + pc-NC group, the expression level of NR2B protein in ICH + pc-CK2 group decreased (*P* = 0.0342; Figure 3D), on the contrary, the phosphorylation level of NR2B at S1480 site increased (*P* = 0.0259; Figure 3D). In addition, the level of NR2B-PSD95 complex decreased (detected by CO-IP; *P* = 0.0163; Figure 3E) and the level of apoptotic protein caspase3 decreased (*P* = 0.0017; Figure 3D). Thus, overexpression of CK2 significantly alleviated the neurological dysfunction in ICH rats, and reduced the neuronal injury in ICH rats by phosphorylating NR2B at S1480 site, down-regulating the expression of NR2B and interfering with the interaction between NR2B and PSD95.

### The overexpression of CK2 enhanced the proliferation and activation of astrocytes and inhibited the apoptosis of neurons

**FIGURE 4:**
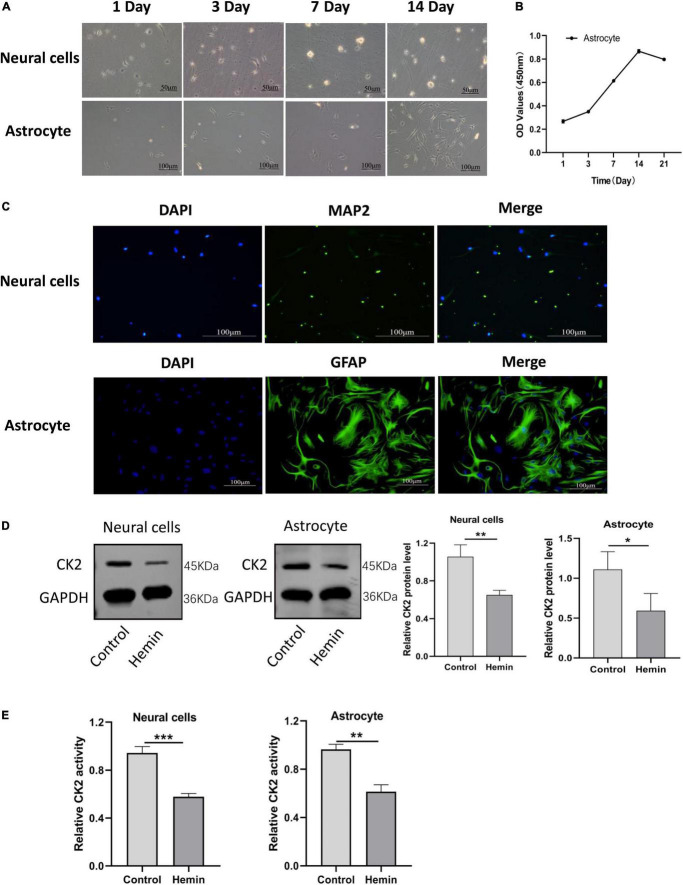
CK2 activity and expression levels were decreased in cultured neurons and astrocytes treated by hemin. **(A)** Morphological changes in neurons and astrocytes were observed. **(B)** Proliferation ability of astrocytes was detected using CCK8 assay. **(C)** Neurons and astrocytes were identified using immunofluorescence. **(D)** Hemin treatment decreased CK2 protein expressions levels in cultured neurons and astrocytes. **(E)** Hemin treatment decreased enzyme activity of CK2 in cultured neurons and astrocytes. The cell experiment was repeated three times independently. Data were expressed as mean ± SD. *p < 0.05, **p < 0.01, ***P < 0.001.

In order to further understand the mechanisms of ICH and to explore effective targets for the treatment, we cultured neurons and astrocytes *in vitro* (Figure 4A). CCK8 experiment showed that with the extension of culture time, the OD value of cells increased, and the proliferation ability of astrocytes reached the peak on the 14th day (Figure 4B). In our cultures, the purity of neurons was more than 95%, and the positive expression of glial fibrillary acidic protein (GFAP) in astrocytes was higher than 95% (Figure 4C). Then, we treated neurons and astrocytes with Hemin, and detected the protein expression of CK2 and the activity of CK2. In the cells treated with Hemin, the expression of CK2 was lower than that of the control cells (*P* = 0.0065; *P* = 0.0439; Figure 4D), and the enzyme activity of CK2 was lower than that of the control cells (*P* < 0.001; *P* = 0.0011; Figure 4E).

**FIGURE 5:**
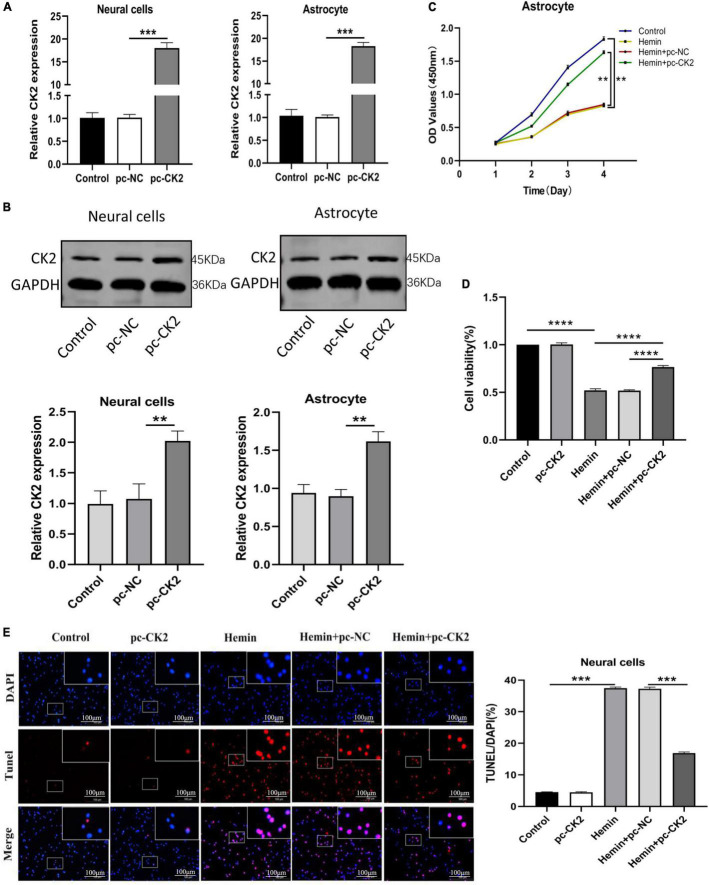
Overexpression of CK2 facilitated the proliferation of astrocytes, and inhibited apoptosis of neurons. pc-CK2 was transfected into Hemin-treated neurons and astrocytes. **(A)** Expressions of CK2 in cells were detected using RT-qPCR. **(B)** CK2 expression was detected using Western blot. **(C)** Proliferation ability of astrocytes was detected using CCK8 assay. **(D)** The viability of neurons was quantified by CCK8 assay. **(E)** Cell apoptosis was measured using TUNEL staining. The cell experiment was repeated three times independently. Data were expressed as mean ± SD. **p < 0.01, ***p < 0.001, ****p < 0.0001.

In order to explore the role of CK2 overexpression in neurons and glial cells, we transfected pc-CK2 into neurons and glial cells treated with Hemin. RT-qPCR and Western Blot were used to confirm the transfection efficiency (*P* < 0.001, Figure 5A; *P* = 0.0052, *P* = 0.0014, Figure 5B). We used CCK8 method to detect the proliferation of glial cells overexpressing CK2 and found that compared with the cells treated with Hemin + pc-NC, the cells treated with Hemin + pc-CK2 had a stronger ability of proliferation (*P* < 0.0001; Figure 5C). Then, we quantified the viability of CK2 overexpression neurons by CCK8 method, and detected the apoptosis of CK2 overexpression neurons by TUNEL staining. The results showed that compared with Hemin + pc-NC treatment, Hemin + pc-CK2 treatment significantly increased viability of neurons (*P* < 0.0001; Figure 5D). Compared with Hemin + pc-NC treated cells, Hemin + pc-CK2 treated cells had lower apoptosis rate (*P* < 0.001; Figure 5E). Thus, CK2 overexpression enhanced the proliferation and activation of glial cells and inhibited neuronal apoptosis.

### The overexpression of CK2 reduced the inflammatory response of astrocytes and the oxidative stress of neurons

**FIGURE 6:**
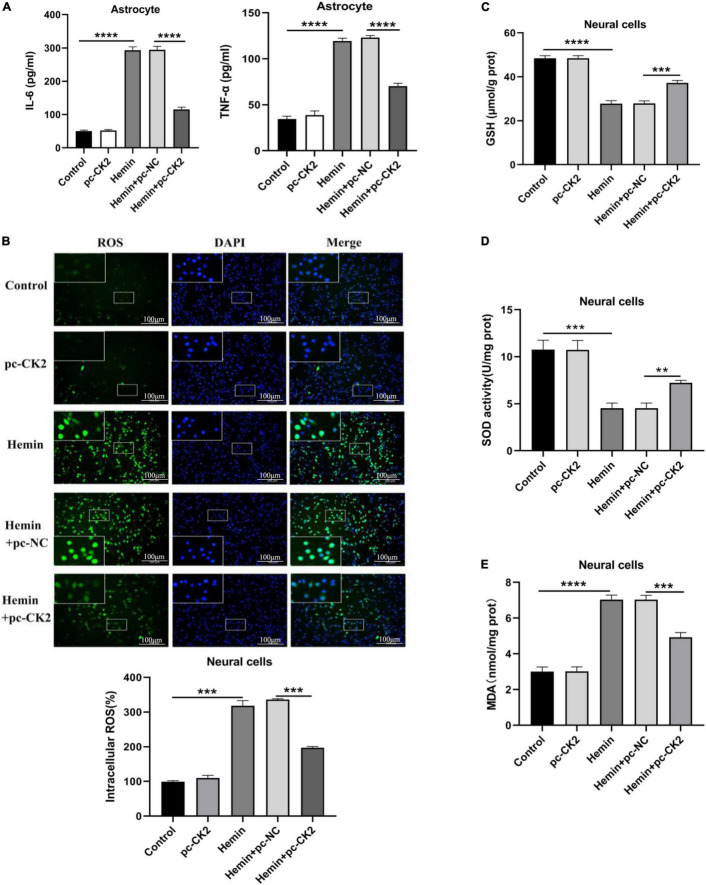
Overexpression of CK2 reduced cellular inflammation and alleviated oxidative stress. pc-CK2 was transfected into Hemin-treated neurons and astrocytes. **(A)** Levels of TNF-α and IL-6 in astrocytes were detected using ELISA. **(B)** Content of ROS was determined by fluorescence probe DCFH-DA. **(C–E)** Contents of GSH, SOD, and MDA were detected using the kits. The cell experiment was repeated three times independently. Data were expressed as mean ± SD. **p < 0.01, ***p < 0.001, ****p < 0.0001.

Neuroinflammation is a promising therapeutic target for hemorrhagic brain injury. We used ELISA to detect the levels of TNF-α and interleukin (IL)-6 in astrocytes. Compared with Hemin + pc-NC treated cells, Hemin + pc-CK2-treated cells showed decreased TNF-α and IL-6 levels (all *P* < 0.0001; Figure 6A). Inflammation could lead to oxidative stress in cells. We used fluorescence probe DCFH-DA to determine the content of reactive oxygen (ROS). After CK2 overexpression, the fluorescence of neurons treated with Hemin was decreased significantly (*P* < 0.001; Figure 6B). In addition, the levels of GSH, SOD, and MDA were detected by colorimetry. Compared with Hemin + pc-NC treated cells, Hemin + pc-CK2-treated cells showed increased levels of GSH and SOD and decreased levels of MDA (*P* < 0.001, Figure 6C; *P* = 0.0016, Figure 6D; *P* < 0.001, Figure 6E). Thus, CK2 overexpression reduced cellular inflammation and oxidative stress.

### CK2 overexpression reduced cell inflammation, apoptosis and oxidative stress by affecting the level of NR2B phosphorylation and NR2B-PSD95 complex

**FIGURE 7:**
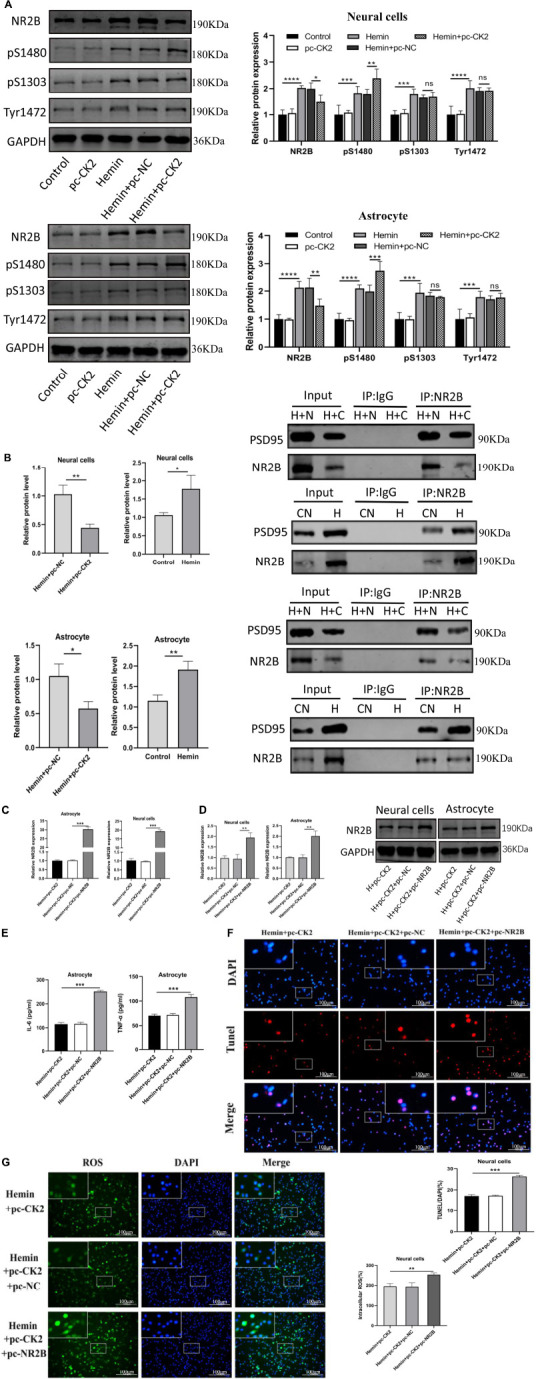
CK2 overexpression reduced cell inflammation, apoptosis and oxidative stress through changing NR2B phosphorylation level and NR2B-PSD95 complex. pc-NR2B was transfected into Hemin + pc-CK2 treated neurons and astrocytes. **(A)** Expression of NR2B, NR2B phosphorylation sites S1480, S1303, and Tyr1472 were determined in cells using Western blot. **(B)** Representative gel images and quantification of co-immunoprecipitation (co-IP) show the level of NR2B-PSD95 complex in cells. **(C)** Expressions of NR2B in cells were detected using RT-qPCR. **(D)** NR2B expression was detected using Western blot. **(E)** Levels of TNF-α and IL-6 in astrocytes were detected using ELISA. **(F)** Cell apoptosis was measured using TUNEL assay. **(G)** Content of ROS was determined by fluorescence probe DCFH-DA. The cell experiment was repeated three times independently. Data were expressed as mean ± standard deviation. **p < 0.01, ***p < 0.001, ****p < 0.0001.

We determined the level of NR2B and its phosphorylation were detected in hemin-treated neurons and astrocytes. NR2B expression and its phosphorylation in Hemin-treated cells was higher than that in control cells (all *P* < 0.001; Figure 7A). Compared with Hemin + pc-NC treated neurons and astrocytes, the expression of NR2B protein in Hemin + pc-CK2 treated neurons and astrocytes decreased (*P* = 0.0414, *P* = 0.0049; Figure 7A), on the contrary, the phosphorylation level of NR2B at S1480 site increased (*P* = 0.0084, *P* < 0.001; Figure 7A). Subsequently, we further detected the level of NR2B-PSD95 complex and found that the level of NR2B-PSD95 complex in Hemin + pc-CK2 group was lower than that in Hemin + pc-NC group (*P* = 0.0040, *P* = 0.0155; Figure 7B). These results suggested that pc-CK2 protect neurons and astrocytes against injury induced by Hemin through affecting the expression of NR2B protein and its phosphorylation level at S1480 site and interfering with the interaction between NR2B and PSD95. In addition, we transfected pc-NR2B into Hemin + pc-CK2-treated cells, and used RT-qPCR and WB experiments to confirm the transfection effect (*P* < 0.001, Figure 7C; *P* = 0.0046, *P* = 0.0029, Figure 7D). Compared with cells treated with Hemin + pc-CK2, the levels of TNF-α and IL-6, apoptosis rate and ROS levels of cells treated with Hemin + pc-CK2 + pc-NR2B were increased (*P* < 0.001, Figures 7E,F; *P* = 0.0083, Figure 7G). Thus, NR2B reversed the inflammatory, apoptotic and oxidative stress responses alleviated by CK2 overexpression after hemorrhagic injury.

## Discussion

Intracerebral hemorrhage is an acute cerebrovascular disease with high morbidity, disability, and mortality. Up to date, the main treatment is to relieve the compression of hematoma on brain tissue, while no effective treatment to improve its prognosis exists (Murthy et al., 2017; Wilkinson et al., 2018). Primary and subsequent secondary brain injuries in ICH are often accompanied by varying degrees of nerve cell necrosis, apoptosis, inflammation and oxidative stress (Lan et al., 2019; Selim and Norton, 2020). The mechanism of nerve cell death and the expression of related regulatory genes remain unclear. Thus, information about pathogenesis including apoptosis, inflammatory response, oxidative stress and the regulatory mechanism of gene expression after ICH may provide new targets for treatment of ICH (Lan et al., 2019; Selim and Norton, 2020).

The brain tissue around the hematoma is subjected to compression and ischemia after ICH, and many cellular and molecular mechanisms are involved in neuronal damage (Lee et al., 2017). Specific mechanisms include the activation of excitatory amino acids and their receptors, persistent neuronal depolarization, release of inflammatory factors and activation of protein kinases (MacLellan et al., 2008). In the clinical samples of cerebral hemorrhage, we found that the expression of casein kinase CK2 was significantly down-regulated. Previous studies have shown that the inactivation of CK2 in mouse brain after cerebral ischemia increases the production of ROSs and the death of neurons by increasing the activity of NADPH oxidase (Kim et al., 2009). CK2 is a key regulator of NADPH oxidase and a neuroprotective agent after brain oxidative stress injury (Kim et al., 2009). The upregulation of synaptic *N*-methyl-D-aspartate receptor 2A (GluN2A) and the increase of NMDAR activity mediated by CK2 are the key factors for the hyperexcitability of hypothalamic paraventricular nucleus neurons (Ye et al., 2012). In addition, CK2 plays an important role in cellular processes, important biological processes and development (Litchfield, 2003; Allada and Meissner, 2005; Moreno-Romero et al., 2008; Seldin et al., 2008; Singh and Ramji, 2008; Dominguez et al., 2009).

We found in ICH rats that CK2 activity in the ipsilateral hemisphere decreased significantly after ICH. In addition, NR2B expression levels were increased and the CK2 expression was decreased in ICH rats, and these changes reached a peak at 24 h after ICH. These data suggests that CK2 plays a regulatory role in hemorrhagic brain injury by affecting NR2B. Subsequent *in vivo* experiments further verified the effect of CK2 on ICH rats. Overexpression of CK2 alleviated neurobehavioral defects, brain water content and neuronal damage in ICH rats (Figures 3B,C). In addition, we found that CK2 overexpression decreased NR2B expression in ICH rats, up-regulated the phosphorylation level of NR2B at S1480 site, and decreased the level of NR2B-PSD95 complex. Furthermore, we found that CK2 overexpression reduced the neuronal apoptosis as indicated by a reduction of apoptotic protein caspase 3. In addition, NR2B protein level and its phosphorylation status at S1480 show the same trend in the ICH rat model (Figure 2C) but respond differently toward CK2 overexpression in ICH rats (Figure 3D). This may be due to the fact that the phosphorylation of NR2B at the S1480 site may be affected by other kinases after cerebral hemorrhage (Chung et al., 2004).

To further determine the role of CK2 in ICH, we established a cell model of ICH by treating cultured primary neurons and astrocytes with Hemin. The expression level of CK2 protein and enzyme activity of CK2 in Hemin-treated cells were lower than in control cells. After ICH, the accumulation of blood leads to the increase of local pressure and the destruction of normal anatomical structure, and neurons are first affected by local compression and insufficient blood flow (MacLellan et al., 2008). Astrocytes are the most abundant cell types in brain tissue. They can provide nutrition for neurons, regulate cerebral blood flow, maintain the blood-brain barrier and regulate the level of extracellular glutamate. During brain injury, astrocytes are activated to produce neurotrophic factors and absorb excessive glutamine, thereby protecting neurons and reducing neuronal damage (Pan et al., 2016). In this study, we transfected lentivirus carrying CK2 gene (pc-CK2) into neurons and astrocytes treated with Hemin. The overexpression of CK2 enhanced the proliferation and activation of astrocytes and inhibited the apoptosis of neurons. It has been reported that inflammation plays a key role in the initiation, progression and recovery of ICH, and anti-inflammatory therapy reduces hemorrhagic brain injury and promote cell survival in ICH (Lee et al., 2017; Lan et al., 2019; Selim and Norton, 2020). In addition, oxidative stress leads to neuronal apoptosis (MacLellan et al., 2008). Thus, inflammation and oxidative stress are effective interventional targets to treat ICH. Recent evidence suggests that CK2 plays an important role in controlling inflammation. For example, in patients with ulcerative colitis and Crohn’s disease, CK2 can promote wound healing by inhibiting apoptosis under inflammatory conditions (Koch et al., 2013). CK2 activity inhibits the inflammatory response of myeloid cells. CK2 deficiency increases recruitment, activation and drug resistance of inflammatory myeloid cells after systemic Listeria monocytogenes (Lm) infection (Larson et al., 2020). In this study, we demonstrated that the overexpression of CK2 could reduce the inflammatory response of astrocytes and the oxidative stress of neurons.

Intracerebral hemorrhage leads to the release of a large number of endogenous molecules, including glutamate, Ca^2+^, thrombin, hemoglobin, iron and IL-6, etc. (Iacobucci and Popescu, 2017; Marques Macedo and Gama Marques, 2020). The rapid increase of glutamate level in damaged brain tissue activates NMDAR to damage neurons. For many diseases, including cerebral hemorrhage, NMDAR plays an important role in excitatory synaptic transmission and plasticity in different brain regions (Lau and Zukin, 2007; Li et al., 2008). Functional NMDAR usually consists of two NR1 subunits and two NR2 (NR2A-D) subunits (Lee et al., 2014). NR2B subunit plays an important role in NMDAR-mediated injury. Several phosphorylated amino acid sites at NR2B subunit terminal can quickly regulate the opening rate of NMDAR and its expression on the cell membrane, and further affect its function. For example, the phosphorylation of NR2B at S1303 site can increase the opening rate of NMDAR and aggravate the neuronal death caused by ischemia (Tu et al., 2010). Phosphorylation of Tyr1472 site interferes with the binding of NR2B and AP2 proteins, resulting in an increase in the expression of NR2B on the cell membrane (Lavezzari et al., 2003). The phosphorylation of NR2B at S1480 site affects the binding of NR2B to PZD domain, which promotes the intracellular transfer of NMDA receptor and down-regulates the expression of NR2B (Chung et al., 2004). In this study, the levels of NR2B and its phosphorylated proteins in both *in vivo* and *in vitro* ICH models were significantly higher than those in the control group. CK2 overexpression treatment could down-regulate the expression of NR2B protein, up-regulate the phosphorylation level of NR2B at S1480 site, and decrease the level of NR2B-PSD95 complex. On the contrary, overexpression of CK2 had no effect on the phosphorylation of NR2B at S1303 and Tyr1472 sites. These results suggest that CK2 protects against hemorrhagic injury by affecting the expression of NR2B protein and its phosphorylation level at S1480 site and interfering with the interaction between NR2B and PSD95. In addition, we overexpressed NR2B in Hemin-pc-CK2-treated cells, and then assessed TUNEL apoptosis staining, ROS levels, and inflammatory cytokines TNF-α and IL-6 levels. As expected, NR2B reversed the cellular inflammation, apoptosis and oxidative stress reduced by CK2 overexpression after hemorrhagic injury.

Our results are the first study to show that CK2 plays a protective role in ICH-induced neuronal apoptosis, inflammation and oxidative stress through the regulation of NR2B phosphorylation. However, the limitation of this study is that we only investigated the regulation of NR2B by CK2. The regulation of CK2 in brain hemorrhage is a complex network involving multiple genes. In this study, we did not determine whether the nuclear factor (NF)-kB or NF-E2 related factor 2 (Nrf2) pathway is involved in the role of CK2 in cerebral hemorrhage. In addition, we did not investigate whether CK2 has other functions in brain hemorrhage, such as improving blood-brain barrier permeability and affecting autophagy. Therefore, we could not determine the possibility that other pathways are involved in the role of CK2 in cerebral hemorrhage, which deserves further investigation in future studies. In addition, the exact mechanism of CK2 effect on NR2B was not elucidated. Whether CK2-mediated phosphorylation of NR2B S1480, is regulated by other kinases or dependent on NMDAR’s own activity needs to be further explored.

## Conclusion

Our study provides new evidence to reveal the role of CK2-mediated NR2B phosphorylation in inflammatory response, neuron apoptosis and oxidative stress after hemorrhagic brain injury. This new information greatly improves our understanding of the molecular mechanism in the pathogenesis of ICH. Our findings indicate that CK2 and NR2B may be new potential therapeutic targets for the treatment of ICH.

## Data availability statement

The original contributions presented in this study are included in the article/supplementary material, further inquiries can be directed to the corresponding author.

## Ethics statement

The studies involving human participants were reviewed and approved by the Ethics Committee of the second Hospital of Hebei Medical University. The patients/participants provided their written informed consent to participate in this study. The animal study was reviewed and approved by the Ethics Committee of The Second Hospital of Hebei Medical University.

## Author contributions

ZS and ZZ designed the study and wrote the manuscript. ZS, QL, XL, YS, CN, QJ, and XW performed the behavioral testing and experiments and analyzed the data. ZS, XL, YZ, and ZZ contributed to revising the manuscript. All authors read and approved the final manuscript.

## Conflict of interest

The authors declare that the research was conducted in the absence of any commercial or financial relationships that could be construed as a potential conflict of interest.

## Publisher’s note

All claims expressed in this article are solely those of the authors and do not necessarily represent those of their affiliated organizations, or those of the publisher, the editors and the reviewers. Any product that may be evaluated in this article, or claim that may be made by its manufacturer, is not guaranteed or endorsed by the publisher.
